# Integration of geriatric assessment-guided care plan modifications and interventions into clinical paths of older adults with cancer (GORILLA): a feasibility approach

**DOI:** 10.1007/s41999-026-01542-7

**Published:** 2026-07-12

**Authors:** Rea Sujin Mayland, Merlin Deterding, Filippo Maria Verri, Sabine Heublein, Alma Aslan, Chantal Flemm, Julia Achilles, Amin T. Turki, Florian Roghmann, Dennis Akuamoa-Boateng, Pia Halagiera, Christian Baues, Sarah Flossdorf, Nuowei Wang, Dirk Strumberg, Gero Lueg, Rainer Wirth, Michael Denkinger, Nina Rosa Neuendorff

**Affiliations:** 1 Agaplesion Bethesda Ulm, Geriatric Centre Ulm/Alb-Donau, Ulm, Germany; 2 Institute for Geriatric Research Ulm, Ulm University Medical Center, Ulm, Germany; 3 Department of Geriatrics, Marien Hospital Herne, University Hospital, Ruhr University Bochum, Herne, Germany; 4 Department of Gynaecology, Ulm University Medical Center, Ulm, Germany; 5 Department of Hematology and Oncology, Marien Hospital Herne, University Hospital, Ruhr University Bochum, Herne, Germany; 6 Department of Urology, Marien Hospital Herne, University Hospital, Ruhr University Bochum, Herne, Germany; 7 Department of Radiation Oncology, Marien Hospital Herne, University Hospital, Ruhr University Bochum, Herne, Germany; 8 Department of Obstetrics and Gynecology, Marien Hospital Herne, University Hospital, Ruhr University Bochum, Herne, Germany; 9 Institute for Medical Informatics, Biometry and Epidemiology, University Hospital, University Duisburg-Essen, Essen, Germany

**Keywords:** Geriatric assessment with management, Comprehensive geriatric assessment, Older adults, Geriatric oncology, Trial, Implementation research

## Abstract

**Purpose:**

A comprehensive geriatric assessment (CGA) followed by geriatric assessment-guided interventions was demonstrated to reduce treatment-related toxicities CTCAE III°–V° in older adults with cancer undergoing systemic cancer treatments, but implementation remains insufficient. Thus, we aimed to evaluate the feasibility of implementing CGA into routine care and multidisciplinary tumor boards (MDTs) in Germany within a bicentric feasibility trial (DRKS00035569; 19.12.2024).

**Methods:**

Patients ≥ 65 years with positive geriatric screening (G8 < 15 points) and all patients ≥ 70 years received CGA as part of their routine care. Results were presented during MDT discussions to derive treatment recommendations. After CGA, patients were asked for trial participation which included data analysis and a telephone follow-up after 3 months. Clinicians participating in the MDT were asked about the added value of CGA presentation. Primary endpoint was the estimation of patient’s willingness to participate with an accuracy of ± 7.5% to inform design for a later (cost) effectiveness trial.

**Results:**

75 patients received CGA (62.5% females). Of those, 72 (96%) agreed to participate (95% confidence interval, [0.8875; 0.9917]). With an accuracy of estimating the willingness to participate of < |7.5%|, the primary endpoint was reached. The median age was 76.7 years (range 69–92 years). A member of the geriatric team attended 2/3 of MDT meetings. Clinicians rated the integration of CGA results predominantly as useful.

**Conclusion:**

Integration of CGA into routine care of older cancer patients is feasible but will likely require adequate geriatric staffing per center. A larger implementation study, evaluating efficacy and cost-effectiveness in the German healthcare system, is necessary.

## Key summary points

**Aim:**

To evaluate the feasibility of implementing comprehensive geriatric assessment into routine care and multidisciplinary tumor boards in Germany within a bicentric feasibility trial.

**Findings:**

The implementation of comprehensive geriatric assessment in cancer care of older adults and integration of geriatric parameters into the multidisciplinary tumor boards was feasible and well appreciated by patients and cancer physicians.

**Message:**

Integrating comprehensive geriatric assessment into routine care should be the standard-of-care for all older adults with cancer.

## Introduction

Outcomes of older patients living with cancer are often worse compared to younger patients. While there are multiple overlapping factors influencing these results, comorbidities and frailty have been shown to complicate treatment tolerability [1].

To address these multifaceted challenges, comprehensive geriatric assessment (CGA), a well-integrated and evidence-based component of general geriatric care, has been introduced into oncology practice [2]. Recently, multiple international studies, such as GAIN [3] and GAP70+ [4] conducted in the USA, INTEGERATE [5] in Australia, and GERICO [6] in Denmark, have shown the benefits of CGA implementation into cancer care. A systematic review including these trials has highlighted that CGA followed by tailored geriatric assessment-guided interventions significantly reduces treatment-related toxicities Common Terminology Criteria of Adverse Events (CTCAE) grade 3–5, while improving Quality of Life (QoL) and increasing the rate of treatment completion [7]. Considering the evidence supporting CGA in oncology, several international guidelines, including the American Society of Clinical Oncology (ASCO) [8] and the European Society for Medical Oncology (ESMO)-International Society of Geriatric Oncology (SIOG) Task Force of Cancer in the Elderly [9], recommend the integration of CGA into cancer care of older adults receiving systemic cancer treatments.

The German evidence-based guideline (“S3-Leitlinie”) on CGA has recently been published and recommends that patients ≥ 65 years with a positive geriatric screening and all patients ≥ 70 years should receive CGA before the start of a new systemic cancer treatment to reduce treatment-related toxicities CTCAE grade 3 or higher [10].

Despite these recommendations and the strong evidence underlining the benefits of CGA in cancer care, the implementation into routine care is yet to be established in Germany like in most other countries worldwide. Availability of CGA for cancer patients is restricted to few local initiatives [11, 12].

To overcome these barriers and assess the feasibility to integrate CGA into routine care of older cancer patients in Germany, we conducted the Integration of geriatric assessment—guided care plan modifications and interventions into clinical paths of older adults with cancer (GORILLA) trial.

## Methods

The study was registered at German Clinical Trials Register (Deutsches Register für klinische Studien, DRKS), DRKS00035569. Details on study design, its rationale, and the respective questionnaires are published as study protocol elsewhere [13]. The study was conducted in accordance with the Declaration of Helsinki and approved by the Institutional Review Board of the University of Ulm on October 30, 2024 (351/24–FSt/Sta), and by the IRB of Westphalia-Lippe on January 23, 2025 (2024-700-f-S).

### Study design and setting

This study was a prospective bicentric feasibility trial performed at the University Hospital Marien Hospital Herne (study center 1) and its respective departments with involvement in cancer care (Urology, Gynecology, Oncology, Geriatrics, Radiation Oncology, and Surgery) and at the University Hospital Ulm (study center 2), Department of Gynecology in cooperation with the Agaplesion Bethesda Clinic, Ulm and the Institute for Geriatric Research of Ulm University.

### Participants

Eligibility criteria: Patients ≥ 65 years with a Geriatric-8 (G8) score ≤ 14 points or ≥ 70 years regardless of their G8 score with newly diagnosed or relapsed cancer prior to multidisciplinary tumor board (MDT) discussion were eligible, if they could give informed consent as evaluated by the study personnel. Intended treatment modalities included radiotherapy, surgery, and systemic treatment approaches (e.g., chemotherapy, targeted therapy, immunotherapy, cellular therapy, endocrine therapy, or combined modalities). Patients with unresectable cholangiocarcinoma, glioblastoma, or acute leukemias were excluded. Patients with basal cell carcinoma or melanoma without intended systemic treatment, or patients who were unable to provide informed consent due to clinically overt dementia and/or language barriers, were also excluded.

### Trial procedures

All patients, regardless of study participation, received a full CGA as part of their routine work-up before their case was discussed during MDT. At center 1, physicians from the oncological departments requested a geriatric consultation, then a geriatric team member approached the respective patient during their in-patient stay based on the consultation request. At center 2, the outpatient calendar was systematically screened, and patients were then approached by a geriatric team member after their outpatient visit. The assessments included in CGA are listed in Table 1 below. Of note, Mini-Mental State Examination (MMSE) was converted into Montreal Cognitive Assessment (MoCA) for better comparability, as recently described [14].

**Table 1:** Assessments included in the comprehensive geriatric assessment

Domain	Assessment
Functional status and mobility	Activities of Daily Living (Barthel) [15] Instrumental Activities of Daily Living [16] SARC-F [17, 18] Short Physical Performance Battery [19] Hand grip strength
Comorbidity	Charlson Comorbidity Index [20] semi-quantitative hearing and/or visual impairment
Social functioning and support	eight questions adapted from ASCO guideline [21–23]
Psychological health	Depression im Alter-Skala (DIA-S) [24] Psychological Distress Thermometer [25]
Frailty	Clinical Frailty Scale (CFS) [26]
Nutrition	Mini Nutritional Assessment Short Form [27, 28]
Cognition	Montreal Cognitive Assessment [22, 29] *or* Mini-Mental State Examination [30, 31]

Geriatric interventions were recommended based on the deficits identified in CGA. Following CGA, patients were asked to consent to trial participation, which included data collection and telephone follow-up after 3 months ± 2 weeks to assess quality of life (QoL), functional status, as well as adherence and/or reception of recommended interventions. The follow-up was intended to obtain a broad overview of patient outcomes (e.g., death, availability by phone) after 3 months, rather than detailed functional trajectories. Patients who declined study participation were documented as not willing to participate without personal identifiers.

A member of the geriatric team presented CGA results during the MDT in the form of a “traffic light” system. This translated into the following: “green” (‘normal functional status/no specific concerns regarding standard treatment’), “yellow” (‘potentially relevant deficit detected, recommended intervention should be considered and standard treatment re-evaluated’), and “red” (‘clinically relevant deficit detected that precludes considered treatment intensity’). Notably, the grading of the traffic light did not reflect a deficit sum score. Instead, it integrated treatment-specific risks for subsequent deterioration based on the identified deficits and the anticipated treatment-related toxicities and was rated individually in relation to the intended treatment regimens. Consequently, even among patients with comparable functional capacities, the traffic light classification could differ significantly based on the anticipated intensity of the cancer treatment (e.g., antihormone therapy for breast cancer might be rated as “yellow” in a frail patient with chronic heart failure, cognitive impairment, and weak social support, whereas polychemotherapy for pancreatic cancer in the same patient might be rated as “red”). Although this categorization was highly individualized and reflected the geriatrician’s subjective clinical judgment, the underlying rationale was discussed for each case. In both study centers, a dual-trained geriatrician-oncologist facilitated these discussions within the geriatric team.

After patient discussion, clinicians (including medical and radiation oncologists, surgeons, urologists, gynecologists, or palliative care specialists) were asked whether the integration of CGA was beneficial to their clinical decision-making, if there were any changes made to treatment recommendations based on the CGA results and which additional geriatric aspects should be included. As only one questionnaire per physician responsible for each patient was provided, the findings reflect the individual views of each physician rather than a consensus of the whole MDT team. After trial completion, selected clinicians were invited for a qualitative interview. Semi-structured interviews were conducted by RSM and NRN. All participants were interviewed individually in German. We used a preliminary semi-structured interview topic guide which was originally in German. The English translation is published elsewhere [13].

### Study objectives

This feasibility trial aimed to inform the design of a future study assessing the efficacy and cost-effectiveness of implementing CGA into German health services by estimating realistic participation rates in the designated study population.

*Primary endpoint*: The primary endpoint of this study is to estimate the willingness of participation with an accuracy of ± 7.5%.

The rationale for this endpoint is that it provides a realistic assessment of the feasibility and informs the design of a confirmatory follow-up trial, including sample size estimation, and suggests strategies to minimize selection bias in this difficult-to-recruit population [13]. This is particularly relevant as frail patients are often underrepresented in clinical research, and even trials specifically designed for older adults have shown large discrepancies in the study population compared to real-world populations [32]. A subsequent trial should minimize patient selection, including non-participation due to refusal, as this would dilute the intervention’s true efficacy and distort estimates of associated costs.

*Secondary outcomes:* The secondary endpoints of this study were grouped into (i) implementation and quality measures, including the completion rate of CGA prior to MDT, attendance rate of a geriatrician during MDT, reasons for non-performance or non-presentation of CGA, and clinician-reported usefulness and influence on MDT recommendations; and (ii) descriptive clinical/contextual measures, including CGA domain distributions, QoL, functional status, and the recommended geriatric interventions and their implementation after the 3-month follow-up.

Further endpoints comprise the evaluation of the impact of CGA integration into MDT discussions on primary surgical/oncological clinicians, as well as their satisfaction.

### Statistical analyses

The statistical analysis was performed with SPSS statistical software (SPSS Statistics for Windows, IBM Corp, Version 31.0, Armonk, NY, USA); calculation of Clopper–Pearson Interval was performed with Epitools (https://epitools.ausvet.com.au/), generation of before-after plots with R (version 4.5.2), using the packages “readxl”, “dplyr”, “tidyr”, “ggplot2”, “rlang”, and “ggtext”.

Categorical variables were expressed as absolute numbers and relative frequencies (%). Continuous and ordinal variables were summarized as median values and ranges (minimum–maximum) for non-normally distributed data and small sample size. Group comparisons were performed using the Mann–Whitney *U* test for continuous variables with a non-normal distribution, and the Pearson chi-squared test for categorical variables. A *P* value of < 0.05 was considered statistically significant.

Qualitative interviews were transcribed verbally and analyzed with MAXQDA (VERBI Software; Consult Sozialforschung GmbH, Version MAXQDA 24 (Release 24.11.0), Berlin, Germany). All interview transcripts were coded by RSM according to Kuckartz’s steps of content analysis [33]. Initially, the transcripts were coded separately, progressing from defined passages to more abstract coding levels, including emerging topics. The codes were subsequently discussed with NRN, and finally with FMV, NRN, and RSM. No discrepancies remained. Quotations were translated and back-translated using DeepL to ensure linguistic accuracy.

## Results

### Recruitment and feasibility measures

**Fig. 1:**
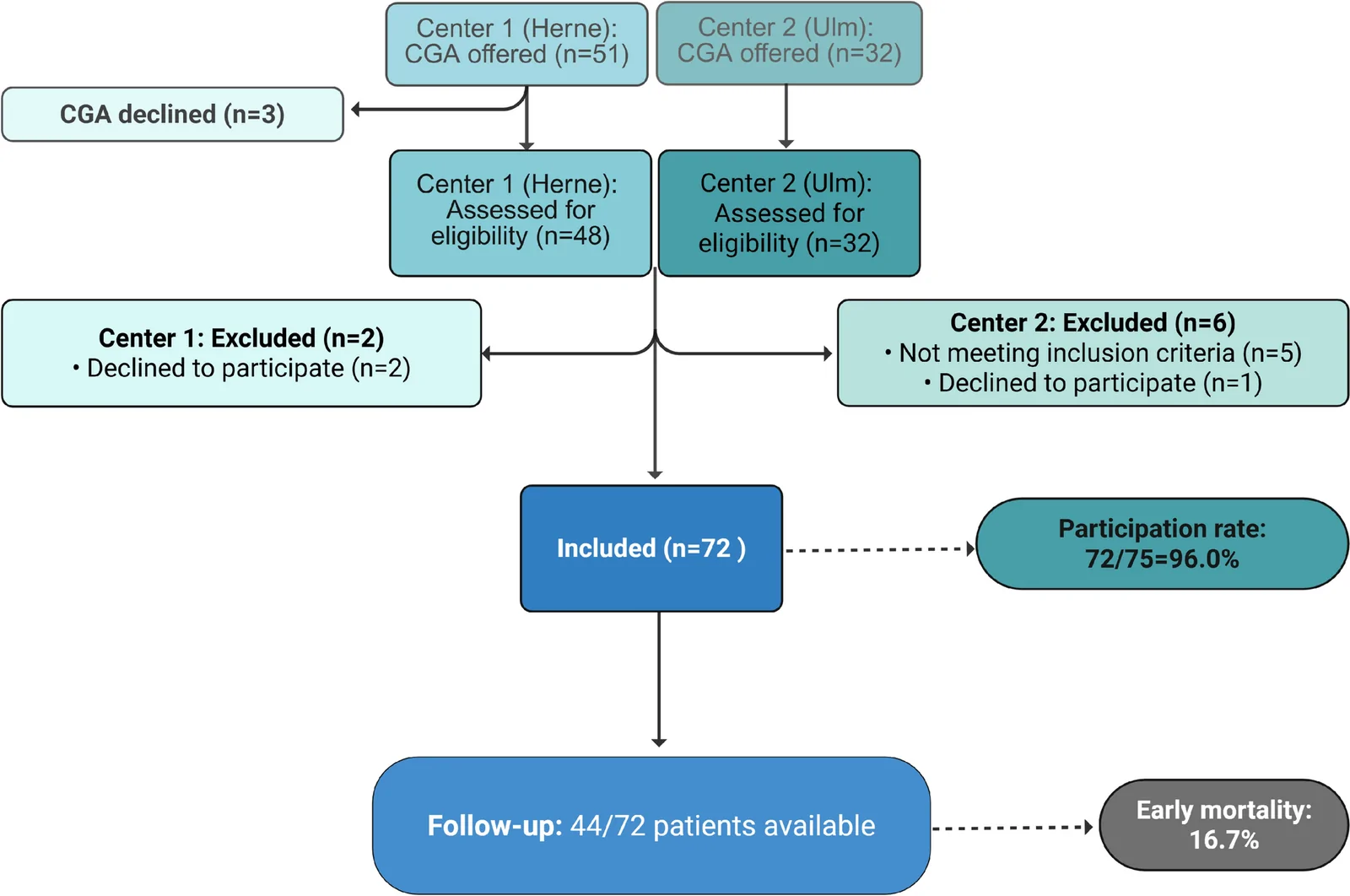
CONSORT diagram

Recruitment time was between February 12 and September 09, 2025 (study center 1, Herne) and between January 24 and June 23, 2025 (study center 2, Ulm). Within this time, CGA was offered to 83 patients as a routine measure which was declined by three patients. Of 80 patients receiving CGA, three declined trial participation. In further five patients, cancer diagnosis or progression was not confirmed. Overall, 72 patients were included, corresponding to a real participation rate of 96% (72/75; 95% CI [0.8875; 0.9917]). As compared with the point estimator, the upper and lower border of the CI is both <  ± 7.5%. Thus, the primary endpoint, to estimate the willingness to participate with an accuracy of ± 7.5%, was reached (Fig. 1).

### Patient characteristics

Patient characteristics are summarized in Table 2. In brief, the median age of the total study population was 76.7 years (range 69.1–92.1 years). The total percentage of patients ≥ 80 years was 38.9% (28/72). Patients from center 1 were primarily recruited as inpatients, whereas study center 2 enrolled outpatients. 62.5% of patients were female with 100% females at center 2 as the recruitment was restricted to the gynecology department. In line, cancer entities differed significantly (see Table 2). The most frequent malignancies at center 1 were bladder, lung, and colorectal cancers, whereas at center 2, patients with cancer of the breast, vagina/vulva, and ovaries were included.

**Table 2:** Patient characteristics

Characteristics	All (*N* = 72)	Center 1 (*N* = 46)	Center 2 (*N* = 26)	*P* value
Median age [years], (range)	76.7 (69.1–92.1)	78.3 (69.1–92.1)	75.0 (70.0–87.0)	0.05
Age ≥ 80 years, *N* (%)	28 (38.9)	20 (43.5)	8 (30.8)	0.288
Gender				
Females, *N* (%)	45 (62.5)	19 (41.3)	26 (100)	< 0.001
Cancer entities, *N* (%)				< 0.001
Lung	9 (12.5)	9 (19.6)	0	
Prostate	1 (1.4)	1 (2.2)	0	
Bladder	11 (15.3)	11 (23.9)	0	
Breast	20 (27.8)	2 (4.3)	18 (69.2)	
Ovarian	5 (6.9)	3 (6.5)	2 (7.7)	
Vaginal/vulva	5 (6.9)	2 (4.3)	3 (11.5)	
Gastric	2 (2.8)	2 (4.3)	0	
Colorectal	8 (11.2)	7 (15.2)	1 (3.8)	
Esophageal	3 (4.2)	3 (6.5)	0	
Endometrial	2 (2.8)	0	2 (7.7)	
Multiple myeloma	1 (1.4)	1 (2.2)	0	
Pancreatic	1 (1.4)	1 (2.2)	0	
Hepatocellular carcinoma	1 (1.4)	1 (2.2)	0	
Cholangiocellular carcinoma	1 (1.4)	1 (2.2)	0	
Lymphoma	1 (1.4)	1 (2.2)	0	
Larynx carcinoma	1 (1.4)	1 (2.2)	0	
Treatment modalities, *N* (%)				< 0.001
Surgery	7 (9.7)	0 (0)	7 (26.9)	
Radiotherapy	8 (11.1)	8 (17.4)	0 (0)	
Systemic treatments (chemo-, immune-, targeted or hormone therapy)	28 (38.9)	25 (54.3)	3 (11.5)	
Multimodal/sequential treatments	23 (31.9)	7 (15.2)	16 (61.5)	
Best supportive care	6 (8.3)	6 (13.0)	0 (0)	
Treatment intention				0.003
Curative intention, *N* (%)	33 (45.8)	15 (32.6)	18 (69.2)	
Palliative intention, *N* (%)	39 (54.2)	31 (67.4)	8 (30.8)	

Curative treatment was intended in 45.8% (*n* = 33), whereas a palliative treatment approach was more common (54.2%, *n* = 39). There was a significant difference regarding treatment intention between both centers. At study center 1, 67.4% of patients (*n* = 31) were intended to receive palliative treatment, whereas at study center 2, only 30.8% (*n* = 8) were considered to undergo palliative treatment.

### Results of the comprehensive geriatric assessment

CGA results are depicted in Table 3. Only a few assessments were incomplete, and none were discontinued due to premature assessment termination. Specifically, hand grip strength data were unavailable in 32 cases, medication review in 6 cases, body mass index (BMI) in one case, and cognitive assessment in three cases (due to severe sensory impairment). In summary, 45.8% of the cohort scored between one and three points on the Clinical Frailty Scale (CFS), indicating that they functioned well prior to their cancer diagnosis. A further 48.6% scored 4–6 points, corresponding from vulnerable to moderately frail, while 5.6% scored higher, displaying severe frailty. Regarding nutrition, 29.2% were screened positive for malnutrition with the Mini Nutritional Assessment-short form (MNA-SF), and 37.5% were at least at risk for malnutrition. 58.3% scored below 9 points in the Short Physical Performance Battery (SPPB), indicating severe sarcopenia-associated functional impairment. 37.5% had a positive screening for depression, and 70.8% had an impaired test result for cognition.

**Table 3:** Results of the geriatric assessment

GA domain	All (*N* = 72)	Center 1 (*N* = 46)	Center 2 (*N* = 26)
G8, median (range)	11 (5–17), *N* = 50	11 (5–17), *N* = 46	14 (9.5–15), *N* = 4
ADL, median (range)	95 (15–100)	92.5 (15–100)	100 (80–100)
IADL, median (range)	7 (0–8)	7 (0–8)	8 (3–8)
DIA-S > 3 points, *N* (%)	27 (37.5)	21 (54.7)	6 (23.1)
Psychological distress, median, (range)	5 (0–10; *N* = 70)	5.25 (0.5–10)	5 (0–10)
CFS, median (range)	4 (1–7)	5 (1–7)	2 (1–5)
CFS, 1–3	33 (45.8)	11 (23.7)	22 (84.6)
CFS, 4–6	35 (48.6)	31 (67.4)	4 (15.4)
CFS > 6	4 (5.6)	4 (8.7)	0 (0)
BMI [kg/m^2^], median (range)	24.2 (15.7–40.9; *N* = 71)	22.8 (16–40.9)	25.8 (15.7–34.9)
MNA-SF < 8 points	21 (29.2)	17 (37.0)	4 (15.4)
MNA-SF 8–11	27 (37.5)	21 (45.7)	6 (23.1)
MNA-SF > 11	24 (33.3)	8 (17.4)	16 (61.5)
SARC-F > 3 points	33 (45.8)	27 (58.7)	6 (23.1)
≥ 1 fall in the last 12 months	22 (30.6)	18 (39.1)	4 (15.4)
SPPB sum score, median (range)	8 (0–12)	6 (0–12)	11 (2–12)
Hearing aid required	29 (40.3)	24 (52.2)	5 (19.2)
Glasses required	69 (95.8)	45 (97.8)	24 (92.3)
MoCA < 26 points	51 (70.8; *N=69*)	42 (91.3; *N* = 45)	9 (34.6; *N* = 24)
MoCA < 20 points	15 (20.8)	13 (28.3, *N* = 45)	2 (7.7; *N* = 24)
Polypharmacy			
Number of medications, median (range)	7 (0–17; *N* = 66)	7 (0–17; *N* = 45)	5 (1–9; *N* = 21)
Number of medications > 3	55 (76.4; *N* = 66)	41 (89.1)	14 (53.8)
Number of medications > 5	42 (58.3, *N* = 66)	32 (69.6)	10 (38.5)
CCI, median (range)	3 (0–12)	4 (0–12)	1 (0–6)

*ADL* activities of daily living, *BMI* body mass index, *CCI* Charlson Comorbidity Index, *CFS* Clinical Frailty Scale, *DIA-S* Depression im Alter-Skala, *IADL* Instrumental Activities of Daily Living, *MoCA* Montreal Cognitive Assessment, *MNA-SF* Mini Nutritional Assessment Short Form, *SARC-F* strength, ambulation, rising from a chair, stair climbing and history of falling; *SPPB* Short Physical Performance Battery

Assessment of medications besides cancer drugs and/or supportive care medication revealed 76.4% of patients (*n* = 55) with more than 3 and 58.3% (*n* = 42) with more than 5 drugs. 21/66 patients (for whom the complete medication record was available) received direct oral anticoagulants (DOACs), 17/66 patients were being treated with aspirin, and 2/66 patients were prescribed phenprocoumon (the most frequently used vitamin K antagonist in Germany, comparable to warfarin). Three of these patients were identified as taking a combination therapy of DOAC and aspirin concurrently. For 18.1% of these patients, a chemotherapy with the potential risk for treatment-related thrombocytopenia was recommended while their active treatment with DOAC, aspirin, or phenprocoumon could potentially increase the risk for bleeding to a greater degree.

Geriatric interventions were recommended based on the identified deficits and included physiotherapy and occupational therapy, modification of the medication, modified management of comorbidities, rehabilitation after certain treatment procedures, evaluation of dysphagia, social support, and cognitive training.

### Functional trajectories

Functional trajectories were analyzed descriptively as contextual feasibility information. Follow-up data after 3 months was available for 44 patients (61.1%). Twelve patients died within the first 3 months (16.7%), eight patients were documented as being alive in the electronic medical records but could not be reached for telephone follow-up, and for another eight patients, the final status remained unknown. Of those, two patients were discharged from hospital to hospice care due to their high symptom burden and their limited life expectancy. Even though we lack their follow-up, we expect that they also died within the 3-month follow-up.

Regarding early mortality, it has to be noted that none of the patients from center 2 died. In contrast, early mortality for patients at center 1 was 26.1% (*n* = 12). One of these patients received treatment in curative intention and one patient received best supportive care without cancer-directed therapy. All patients who deceased had a baseline CFS of 4–6 points. In addition to CGA, 10/46 patients at center 1 received a palliative care consultation. Patients who deceased early received such a consultation in 50% (6/12 cases). Of note, palliative care consultations were restricted to patients with cancers that were regarded as incurable and were not routinely provided as supportive care measures during treatment with curative intent.

**Fig. 2:**
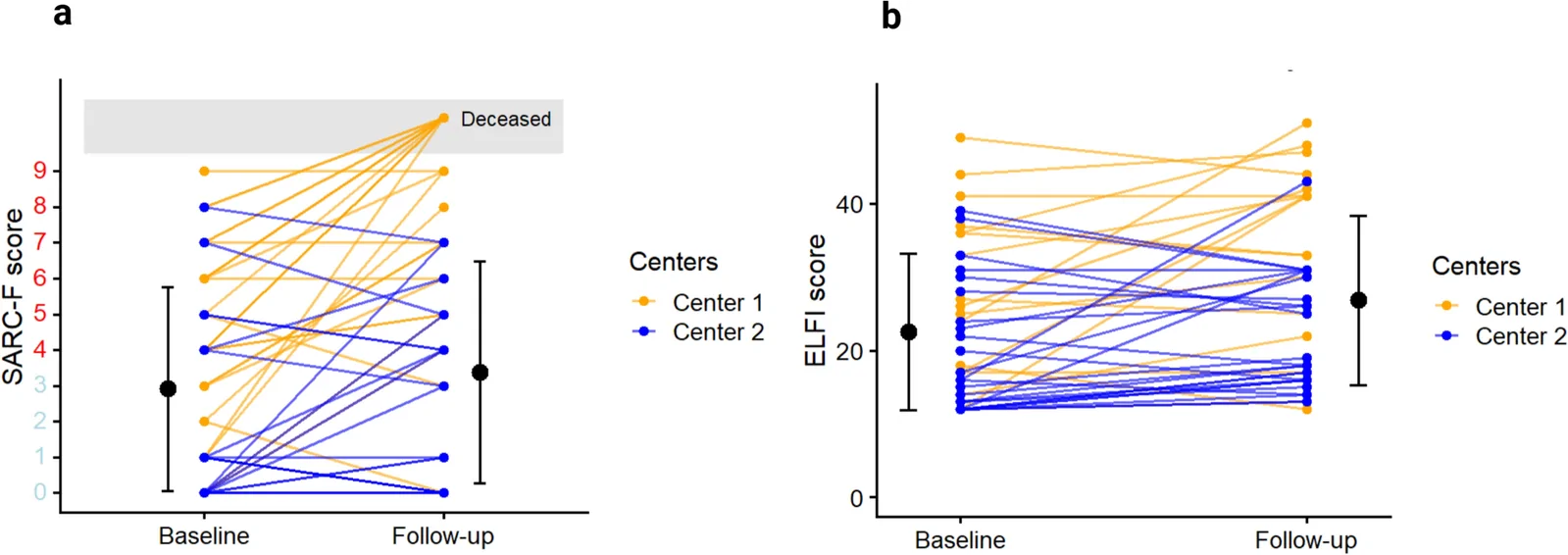
Functional trajectories after 3-month follow-up. **a** Trajectories of SARC-F score during follow-up; **b** trajectories of quality of life measured with ELFI score. Deceased patients or patients lost to follow-up are not included

Functional trajectories including Strength, Assistance with walking, Rise from a chair, Climb stairs and Falls (SARC-F) questionnaire, and The Elderly Functional Index (ELFI) scores over time are depicted in Fig. 2.

### Implementation of CGA results into MDT presentations

#### Feasibility of geriatric MDT presence

**Fig. 3:**
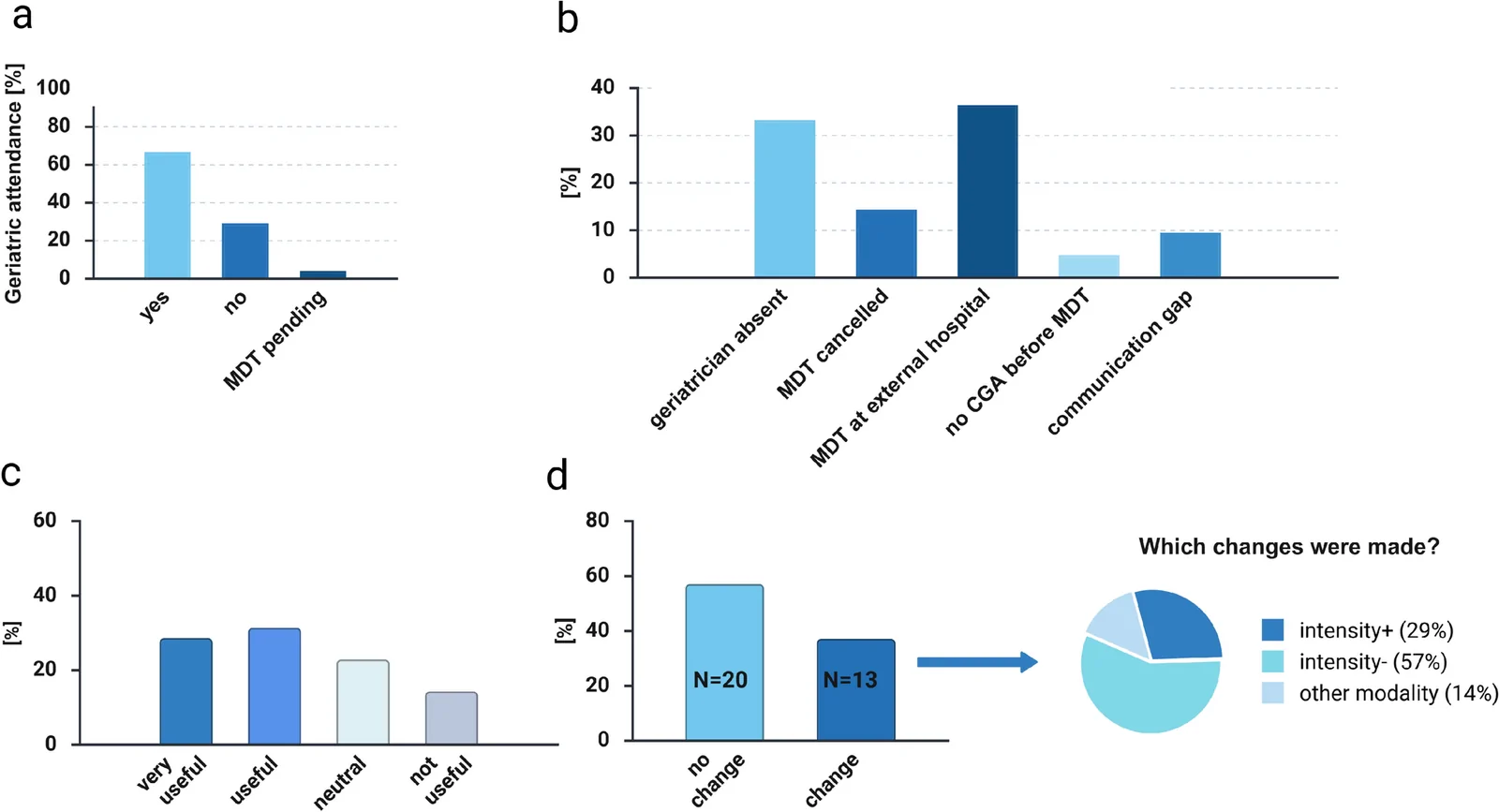
Quantitative assessment of clinicians’ satisfaction with CGA presentation during Multidisciplinary Tumor Boards (MDTs). **a** Geriatric attendance at MDTs; “MDT pending” refers to cancelation or postponing the MDT over several weeks; **b** reasons for cancelation of an MDT and non-attendance of the geriatric team member; **c** clinicians’ answers to “Did you find the integration of CGA into the current patient discussion during MDT useful?”; **d** changes of treatment recommendations during MDT based on CGA results

In total, two-thirds of MDTs were attended by geriatric team members (Fig. 3a). At center 1, 52.2% of MDTs and, at center 2, 92.3% were attended. Of note, at study center 1, only one geriatric team member was available to participate in the MDTs due to structural reasons, whereas at study center 2, four members were available. Reasons for a patient not being discussed at the MDT or for non-attendance of a geriatric team member are depicted in Fig. 3b. In 38.1%, the reason not to present the patient case and CGA results at the MDT was that the patient case had already been discussed at an MDT of another hospital before but without a detailed treatment recommendation. As this was usually not known at the time of study inclusion, it did not influence trial participation. For detailed treatment recommendations, these cases were usually discussed between two clinicians (radiation oncologist, surgeon, medical oncologist) together with the geriatric team member under consideration of CGA results (informal “mini MDT”).

#### Quantitative evaluation of CGA implementation

After each patient discussion for whom CGA was presented at MDTs, the clinicians in charge received an evaluation form. 35 evaluations were available for analysis. Questionnaires were completed by gynecologists (51.4%), radiation oncologists (14.3%), medical oncologists (20%), pulmonologists (11.4%), and palliative care specialists (2.9%). Overall, 11.4% of those had professional experience of less than 6 years (residents in training), 34.3% between 5 and 10 years, 40% 11–20 years, and 14.3% more than 20 years of experience. 60% of clinicians rated CGA implementation as helpful or very helpful (Fig. 3c). In addition, they stated that CGA led to a modification of treatment recommendations in 37.1% of cases. Of those, 28.6% were adjusted to a more intensive treatment recommendation, 57.1% to a de-escalation, and in 14.3% to a different treatment modality (Fig. 3d). It should be noted that the geriatricians did not directly provide treatment recommendations or dose adjustments; rather, the relevant treatment modifications were made by the primary treating medical or radiation oncologists based on the CGA findings. The extent to which MDT recommendations were implemented in clinical practice was not assessed. Notably, the perceived usefulness of the CGA implementation differed significantly between study centers (*P* < 0.001), with physicians at center 1 reporting a benefit in 16/17 cases, compared to 5/18 cases at center 2. CGA implementation led to a change of treatment recommendations in 2/18 cases at center 2 and in 11/17 cases at center 1.

When asked about additional information the clinicians would have liked to receive, they stated in two cases that they would have been interested in detailed recommendations regarding deprescribing/polypharmacy management. In five cases, recommendations for the cancer treatment itself were inquired. In two cases, additional information to estimate life expectancy besides cancer-related mortality, and a detailed recommendation for the management of comorbidities were requested.

#### Qualitative evaluation of CGA implementation

Four interviews with clinicians from both study centers working at consultant level were performed after termination of trial recruitment. One gynecologist, one radiation oncologist, one palliative care specialist, and one medical oncologist were interviewed. Two had participated in at least 2–4 MDTs with presented CGAs, two in more than ten MDTs.

All participants found the implementation of CGA useful, though one participant noted that CGA results did not further influence treatment recommendations in very fit or very frail patients as those were already adjusted based on the clinical presentation. Local challenges of CGA implementation as partially discussed in the interviews are not reported here as it might be of interest only for site-specific management. All but one participant identified mobility, cognition, and social circumstances as the most informative domains of the geriatric assessment. One participant underlined the importance of integrating patient priorities into the recommendations and pinpointed that this aspect is not routinely listed among the CGA results:

“*This is a tumor biology conference, not an actual tumor conference where the focus is on the patient's needs. […] – What are the goals? What are the wishes [the patient] wants to achieve? And with this in mind, it is essential to carry out an assessment in addition to what they collect in terms of the patient's physical and psychological functionality or mental capacity*.” [Interview participant 1]

One participant reported that CGA results led to both escalation and de-escalation of treatment recommendations:

“*So this [the CGA; remark] has been groundbreaking in that it has led to more intensive therapy—even with good tolerability—in fit patients who would otherwise have received de-escalation treatments that would have been more palliative in nature. On the other hand, it has also enabled a critical look at alternative treatment concepts, alternative systemic therapy options, and, in selected cases, certainly also relevant therapy de-escalation*.” [Interview participant 3]

Another participant commented on the effect of CGA implementation as follows:

“*Much of what is otherwise discussed [without implementation of CGA; remark] remains in the realm of opinion, or of postponing or delaying treatment decisions*.” [Interview participant 2]

## Discussion

In this prospective bicentric feasibility trial, we evaluated the feasibility to integrate CGA into the routine care of older adults with cancer according to the German Guideline on CGA. The guideline recommends CGA prior to systemic cancer treatment for all patients ≥ 70 years and for those aged 65–69 years with a G8 score ≤ 14 points [10]. Given the paucity of practicing geriatricians in Germany, we doubt that the majority of cancer centers would be able to ensure geriatric participation on a regular basis at their MDTs. Alternatively, a written summary of CGA results and their implications for cancer treatment provided to the oncologists prior to the MDT could perhaps substitute for direct geriatric attendance. Nonetheless, implementing such an approach would require an intensive educational effort and close collaboration between oncological disciplines and geriatrics to establish an effective and practicable workflow. In addition, geriatric screening is increasingly being mandated for the certification of German cancer centers. If this will translate to a higher demand for subsequent geriatric consultations remains to be seen.

In light of the above, the main contribution of this manuscript was not to re-demonstrate the clinical efficacy of CGA, but to operationalize how CGA can be delivered before MDTs, communicated within MDTs, and used to inform subsequent trial design under routine-care conditions.

It is well described that older and especially frail adults are underrepresented in many clinical trials [34]. These challenges are not limited to trials on cancer-targeting agents but also extend to other interventional trials. A recent report compared the degree of frailty in patients referred to an oncogeriatric outpatient service to patients from the Canadian 5C Randomized Controlled Trial that investigated the effect of CGA on QoL in older cancer patients. Trial participants were significantly younger and had fewer comorbidities, better physical function, nutritional status, and cognition [32]. This demonstrates that even trials dedicated to frail older adults are often not representative of this vulnerable group of patients which limits the extent to which the results can be generalized.

As we aim to design a cost-effectiveness trial of CGA implementation to legitimize the costs for CGA on a population-wide level, the patient cohort must be representative of the general population in such a case. Thus, we prioritized the estimation of willingness to participate as an endpoint for this difficult-to-recruit population to design such a trial. The participation rate was remarkably high with 96% of patients willing to participate. A hypothesis for this high participation rate is that the time spent with and the interest in the patient from the study personnel while conducting CGA as a routine measure prior to informed consent has built deep trust with the patients and alleviated concerns regarding the formalities of the informed-consent process. If this is confirmed in other trials, performance of CGA before consenting and randomization could be a valid option in trials using CGA to tailor treatment intensities and other procedures.

We observed significant differences in terms of frailty and other patient characteristics between both centers which were likely related to the different settings. Study center 1 recruited mainly inpatients, whereas study center 2 recruited outpatients. This led mainly to significant differences in the frailty status between both centers which further influenced other CGA results, treatment intent, and mortality. Of note, statistical differences for patient characteristics were mostly left unreported as many of those are influenced by the setting and the degree of underlying frailty. These differences with the high degree of frailty and related functional deficits clearly demonstrate the need for in-patient care in selected cases despite the ongoing political efforts to deliver cancer care nearly exclusively in the outpatient setting.

In addition, we need to underline that the CFS was applied according to its original definition (functional status 2 weeks prior to presentation). Many cohort trials in oncogeriatrics, particularly retrospective cohort studies, do not adhere strictly to this definition, thereby limiting comparability between cohorts. However, cancer itself can lead to a functional decline, e.g., caused by weight loss, concomitant anemia, or other disease-related complications. Consequently, a timeframe extending the original 2-week window might better reflect the premorbid functional status. Validation of the CFS regarding its prognostic utility when applied to, e.g., a 3-month window, along with other tools might be a valuable tool in the future.

Integrating CGA results into the MDT meetings changed treatment recommendations in 37.1% of the cases. Of note, although the recommendation was changed toward a less-intensive treatment approach in the majority (57%) of cases, a more intensive approach was chosen in 29%. This is in line with a comment by one of the interviewed clinicians who stated that the integration of CGA influenced treatment decisions bidirectionally: it supported escalation to more intensive therapy in fit patients who might otherwise have received less-intensive approaches, while also prompting critical reassessment and de-escalation in cases where vulnerabilities were identified. Under- and overtreatment are equally problematic in geriatric oncology as both extremes can negatively impact survival [35]. Thus, a thorough assessment of the patient’s intrinsic capacity by CGA can support the clinicians to critically reassess their treatment recommendations. The magnitude of changes observed is in line with a recent systematic review describing a change in treatment recommendations based on CGA results in 31% (range 6–56%), mainly toward a less-intensive approach (median 73% of changes) [36]. Another analysis demonstrated that patients who were discussed at an MDT dedicated to geriatric oncology with the respective integration of CGA resulted in 74% of treatment recommendations that matched with the finally perceived treatment [37]. Importantly, in our trial, the observed changes refer to MDT recommendations rather than confirmed changes in the actually delivered treatment, which represents an important limitation of this feasibility study. Since many patients received treatments outside the respective centers, we are not aware of the treatments received and cannot compare those. In addition, statements on changes regarding treatment recommendations were only available in 35/72 cases, which was mostly due to the absence of the clinician who had previously seen/evaluated the patient. This represents an important limitation of our findings. It would have been of interest to compare changes in treatment recommendations based on CGA between physicians already familiar with the patient and those without previous clinical knowledge of the patient, but this was not included in the study protocol.

Despite the reported changes in treatment recommendations, only 60% of clinicians rated the CGA as useful. This is caused by a center effect as most clinicians at center 1 rated CGA as useful compared to a minority at center 2. Most likely, this can be explained by the overall higher fitness of patients at center 2 leading to less conflicting treatment decisions. This is underlined by a statement of an interviewed gynecologist at center 2:

“*In principle, it [the CGA; remark] is helpful. However, many of our patients are quite fit. And then the information gained from the geriatric assessment was often not that significant. Or if the opposite was true and the patient did not appear to be fit and the question was, for example, about chemotherapy, then we often made a similar clinical assessment without necessarily conducting the geriatric assessment. Thus, it wasn't often that this assessment had a major influence on our recommendation.”*

In summary, nevertheless, modification of MDT recommendations represents a proximal and clinically meaningful implementation outcome, reflecting integration of CGA into decision-making processes rather than a clinical efficacy endpoint.

The role of a geriatric team member in the MDT is not yet defined. Clinicians explicitly requested specific recommendations regarding cancer treatment regimens and intensity in five cases. At first glance, this is surprising, given that most geriatricians lack formal training in oncological sub-specialties. One possible explanation is that clinicians seek more robust evidence on dosing and therapy modifications tailored by CGA results. Very few clinical trials have evaluated different treatment regimens or dosages adapted to frailty and CGA in a randomized design. One notable example is the Frailty-adjusted therapy in Transplant Non-Eligible patients with newly diagnosed Multiple Myeloma (FiTNEss) trial [38]. Extending similar trial concepts to other malignancies represents a major challenge in the upcoming years. In clinical practice, a complete integration of geriatrics into standard oncological care with a short but substantial discussion between the two specialists would likely be the preferred, yet logistically demanding approach. As our dynamic traffic light system has not been validated and remains highly individual, a cross-study application involving comparisons remains challenging so far. However, since we still consider this approach to be intuitive and viable, grading the treatment intensity of common treatment protocols based on their tolerability for frail patients with defined geriatric vulnerabilities—developed through an international Delphi consensus—is an option we would like to evaluate in the near future.

The comparatively high mortality rate at center 1, along with the corresponding CGA-identified deficits, suggests that early CGA could help stratify patients at risk or with substantial tumor burden, guide treatment intensity, and prompt an early referral for integrated supportive and specialist palliative care. A recent call for oncogeriatric palliative care emphasized that screening/assessment at the time of diagnosis can stratify and objectivize functional reserve, distinguish tumor-driven from pre-existing frailty, and identify candidates for best supportive care strategies to minimize overtreatment, prevent avoidable treatment-related toxicity, and reduce financial and time burdens [39]. During follow-up, a relatively high mortality among in-patient participants was revealed. As this was at least partially unexpected, this finding has already impacted our routine care, as we now conduct palliative evaluations even earlier, based on cancer-specific prognosis and frailty. Early palliative review and targeted symptom management may further optimize patients prior to treatment and mitigate treatment-related harm [39]. In line with this, our interviews suggested that CGA supports more timely MDT decisions. Despite that, integration of patient priorities besides objective results of the geriatric assessment were requested during one interview and may help earlier transitions to best supportive care pathways when appropriate or highlight treatment alternatives aiming solely at control of cancer-related symptoms to improve quality of life. Although we assessed patient priorities during CGA and often summarized those at the MDTs, this manuscript does not evaluate the impact and associations of patient priorities which will be published separately elsewhere.

Nonetheless, this trial has some limitations. First, we did not assess toxicities as the main outcome measure, which were improved in the previous randomized trials. As the focus was on feasibility, the study cohort was very heterogeneous, limiting comparability regarding efficacy. Accordingly, the observed functional trajectories, intervention uptake, and early mortality should be interpreted as feasibility and trial-design signals rather than as evidence of CGA effectiveness. We also decided not to report frequencies of individual geriatric interventions, because recommendations were highly context-dependent and influenced by pre-existing involvement of geriatrics, palliative care, and other services. Secondly, trial inclusion relied on referral by the clinician in charge rather than a standardized screening of all older cancer patients treated at the respective departments, potentially causing a selection bias toward patients with higher degrees of frailty, at least at center 1. This was also noted by one of the interviewed clinicians:

“*From my point of view, it's less about specific factors and more about [CGA; remark] being implemented in principle. In other words, the systematic nature of the implementation and the avoidance of bias by giving preference to certain patients for screening*.”

Another limitation is the limited availability of patients who responded to the telephone follow-up. Although the high mortality rate was a major factor for being lost to follow-up, we lack detailed data on eight patients who were last documented as being alive in the electronic medical records, as well as six further patients without any information regarding the current status. We know that the re-hospitalization rate is high in older patients with cancer and would hypothesize that at least some patients did not respond due to a competing in-patient stay at another hospital. Furthermore, many patients did not own a mobile phone and provided the landline number for follow-up. Together with a high rate of hearing loss, this might have also contributed to missed follow-ups. For a subsequent larger trial, we do not consider such a follow-up to be feasible and plan another approach.

The lack of patients’ perspective as compared to the physicians’ view has presented itself as an important limitation. The integration of CGA results into informed-consent conversations has a strong potential to support shared decision-making and patient engagement [40]. In a similar implementation trial from Switzerland, 73.7% of responding patients rated CGA-based recommendations as useful and thought that they had benefited from the geriatric appointment [41]. In a subsequent trial, we wish to further evaluate the patients’ perspective to better understand its importance in shared decision-making and patient engagement.

For further implementation in routine care, standardized screening should be implemented to minimize possible bias and to enable all patients to receive specialized oncogeriatric care. A limited number of CGA ratings by clinicians after the MDTs and qualitative interviews were available, limiting the validity of the findings. A selection bias cannot be excluded. Finally, we excluded patients unable to give consent due to overt dementia. Despite that, we observed a high percentage of cognitive deficits, especially at center 1.

## Conclusions

The integration of CGA into the routine care of older adults with cancer is feasible if appropriate personnel are available. A subsequent trial on cost-effectiveness is required to foster further implementation of CGA in the German healthcare system. In addition, future implementation studies should not only assess functional domains but also systematically integrate patient priorities and goals of care into CGA-guided MDT discussions.

## Data Availability

Data are provided at a reasonable request to the corresponding author. This manuscript was posted as preprint at the following link: https://www.preprints.org/manuscript/202601.0523.
